# EV-origin: Enumerating the tissue-cellular origin of circulating extracellular vesicles using exLR profile

**DOI:** 10.1016/j.csbj.2020.10.002

**Published:** 2020-10-14

**Authors:** Yuchen Li, Xigan He, Qin Li, Hongyan Lai, Hena Zhang, Zhixiang Hu, Yan Li, Shenglin Huang

**Affiliations:** a Department of Integrative Oncology, Fudan University Shanghai Cancer Center, and the Shanghai Key Laboratory of Medical Epigenetics, the International Co-laboratory of Medical Epigenetics and Metabolism, Ministry of Science and Technology, Institutes of Biomedical Sciences, Fudan University, Shanghai 200032, China; b Department of Oncology, Shanghai Medical College, Fudan University, Shanghai, China; c Department of Hepatic Surgery, Fudan University Shanghai Cancer Center, Shanghai Medical College, Fudan University, Shanghai 200032, China

**Keywords:** CSF, cerebrospinal fluid, exLR-seq, extracellular vesicles long RNA sequencing, EVs, extracellular vesicles, exLRs, extracellular vesicles long RNAs, GEP, gene expression profile, TSGs, tissue-specific genes, TSS, tissue-specific score, TPM, transcripts per million, Circulating EVs, Extracellular vesicles long RNA sequencing, Tissue-cellular origin, Tissue-specific genes

## Abstract

Extracellular vesicles (EVs) are complex ecosystems that can be derived from all body cells and circulated in the body fluids. Characterizing the tissue-cellular source contributing to circulating EVs provides biological information about the cell or tissue of origin and their functional states. However, the relative proportion of tissue-cellular origin of circulating EVs in body fluid has not been thoroughly characterized. Here, we developed an approach for digital EVs quantification, called EV-origin, that enables enumerating of EVs tissue-cellular source contribution from plasma extracellular vesicles long RNA sequencing profiles. EV-origin was constructed by the input matrix of gene expression signatures and robust deconvolution algorithm, collectively used to separate the relative proportions of each tissue or cell type of interest. EV-origin respectively predicted the relative enrichment of seven types of hemopoietic cells and sixteen solid tissue subsets from exLR-seq profile. Using the EV-origin approach, we depicted an integrated landscape of the traceability system of plasma EVs for healthy individuals. We also compared the heterogenous tissue-cellular source components from plasma EVs samples with diverse disease status. Notably, the aberrant liver fraction could reflect the development and progression of hepatic disease. The liver fraction could also serve as a diagnostic indicator and effectively separate HCC patients from normal individuals. The EV-origin provides an approach to decipher the complex heterogeneity of tissue-cellular origin in circulating EVs. Our approach could inform the development of exLR-based applications for liquid biopsy.

## Graphical abstract

**Figure:**
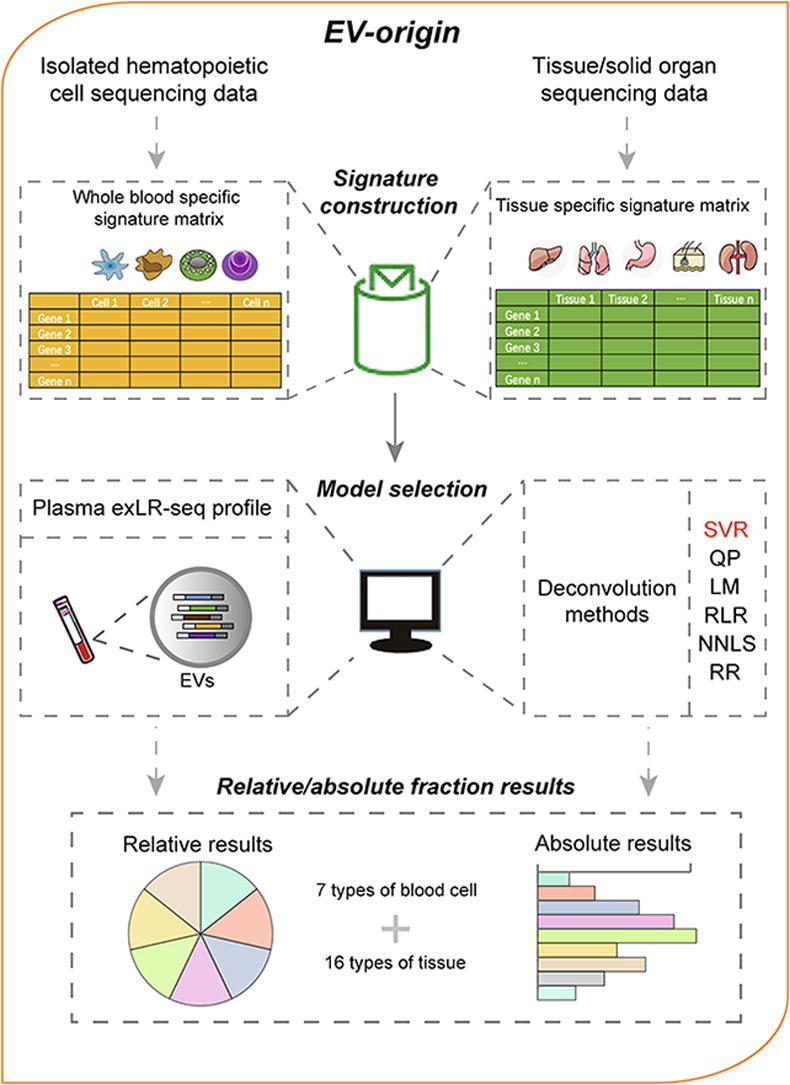

## 1 Introduction

Extracellular vesicles (EVs), which include exosomes and microvesicles, are nano-scaled and membrane-enclosed particles released from essentially all eukaryotic cells [1]. EVs contain proteins, lipids, and nucleic acids that are delivered from the parent cells to the recipient cells [2]. These bioactive molecules function as mediators of intercellular communication [3], [4]. EVs are associated with most pathological conditions, including cancers, cardiovascular diseases, neurologic disorders, and infectious diseases. These particles also served as diagnostic biomarkers, therapeutic targets, and medicine carriers for disease therapeutics [5]. Recent evidence suggests that body fluid EVs are involved in numerous physiological processes and play essential roles in remodeling homeostasis of the body [6]. In particular, plasma EVs originate from host cells mediate their mutual regulation locally or remotely, these EVs have cell type-specific biomolecules and could be exploited as predictive biomarkers for disease treatment [7], [8], [9], [10], [11], [12], [13], [14]. The brain cell originated EVs could also be detected in CSF reflect the physiological and pathological changes taking place in the originated brain tissue [15], [16], [17]. Meanwhile, EVs encapsulate RNAs reflect the phenotype and functional states of their parent cells [18]. Tracing the source of tissue-specific genes (TSGs) in circulating EVs long RNA (exLR) pool is a noninvasive strategy for early detection and therapeutic evaluation of human diseases [19], [20].

EVs in plasma are heterogeneous, originating from different cell types and from diverse sources, which limits the utility of bulk EV analysis methods. Single-particle measurements such as flow cytometry would be preferred to trace tissue-cell-originated EVs by given cell surface receptors and other biomolecules [21]. Of note, the flow cytometry-based approaches play an important role in understanding the origins, functions, and diagnostic and therapeutic significance of EVs in health and disease [22], [23]. Several studies have used flow cytometry analysis to trace platelet- and lymphocyte-derived EVs in circulation, these results indicated plasma EVs predominantly originated from platelets, erythrocytes, and other leucocytes [24], [25], [26], [27], [28]. While the EVs sorting techniques have been developed and applied clinically, comprehensive assessment of the heterogeneity of multiple tissue-cellular origins for circulating EVs remains challenging.

Recently, a number of programs have been developed to digitally estimate tissue-cellular constituents from mixture transcriptional profiles using robust deconvoluting algorithms. In particular, these programs have been used to trace the fraction of immune cells from tissue transcriptional data [29], [30], [31], [32], [33]. The basis of the hypothesis for these strategies is that the gene expression profile in an admixture is a linear combination of the genes specifically enriched from all the included cell types [34]. Several machine learning methods have been reported, include Ridge Regression (RR), Robust Linear Regression (RLR), linear least square regression (LLSR), quadratic programming (QP), Nonnegative Least Squares (NNLS), and support vector regression (SVR). These deconvolution models are used to infer the enrichment heterogeneity of cell types from the gene expression data [31], [32], [35], [36], [37], [38]. Meanwhile, the computational measures have enabled the assessments of the source contribution of tissue-cellular components from other types of sequencing data.

We previously developed a genome-wide analysis of exLRs termed exLR sequencing (exLR-seq) among healthy individuals and cancer patients [19]. A large amount of TSGs was highly expressed in healthy plasma exLR-seq transcriptomes and showed diverse enrichment levels in multiple disease conditions. However, compared with the tissue transcriptomes, the expression pattern of these TSGs differed in plasma EVs and only a fraction of these genes showed tissue-cellular traceability in circulation. These results indicated the prediction system based on tissue bulk data is not reliable on biofluid EVs. Thus, it is necessary to develop an optimal strategy that is independently suitable for the deconvolution scenario of exLRs profiles. Investigating the idealized input matrix of reference gene expression signatures and benchmarking them with viable mathematical algorithms according to types of exLRs sequencing data is a potential strategy to resolve this deconvolution problem for circulatory EVs.

In this study, we developed a computational method to portray the relative and absolute tissue-cellular enrichment results of plasma EVs from exLR-seq profiles. We also explored and compared the distinct tissue-cellular origins from plasma EVs samples with diverse disease conditions. Comparison with healthy individuals revealed the upregulation of the hepatogenic fraction associated with hepatic disorder and accurately predicted the development and progression of liver disease, especially for hepatocellular carcinoma (HCC). We provided our source code to the community and hope that this repository will allow investigators to acquire a better perspective of the complex heterogeneity of tissue-cellular origin in biofluid EVs.

## 2 Materials and methods

### 2.1 Sample collection for exLR-seq

The plasma exLR-seq samples were collected in our previously published study. They included healthy individuals (n = 101), patients with HCC (n = 71), benign hepatic tumor (n = 18), hepatitis (n = 5), hepatic cirrhosis samples (n = 8), gastric cancer (GC, n = 9), colorectal cancer (CRC, n = 12), breast cancer (BRCA, n = 10) and kidney cancer (KIRC, n = 15). The validation cohort comprised healthy (n = 14), hepatic benign disorder (n = 6), and HCC samples (n = 33). We have summarized the sample’s number with their demographic and clinical characteristics of entire cohorts in a diagram and presented it as Table. S5 in supplementary material. Four plasma-serum paired samples from one healthy individual were also included in this study, three of plasma samples collected in EDTA tubes were stored for different durations (0, 2, and 8 h) at room temperature (15–25 °C) and processed within 2 h. The remaining serum sample was allowed to stand at 37℃ for 30 min before sample processing. Two 1–2 ml cerebrospinal fluid samples were collected from patients with neurological disorder in this study. All the CSFs were sampled by needle aspiration from the lumbar subarachnoid space. We used our previously published exLR-seq method to carry out EVs purification, EVs-RNA isolation, and RNA-seq library preparation, respectively [19].

### 2.2 The tissue-cellular traceability system of plasma EVs

#### 2.2.1 Filtration of TSGs and construction of signature matrices

We constructed two representative signature matrices to deconvolute the blood and tissue fractions from exLR-seq transcriptomes. The expression profiles of 31 types of solid organic tissues (TPM quantification with gencodeV23 annotation) were downloaded from the GTEx portal (https://gtexportal.org/). We used a tissue-specific score (TSS) strategy, with scores ranging from 0 to 5 for each gene from expression atlas of the GTEx portal [19], [39].

To construct the reliable tissue signature matrix and minimize the influence of other unknown constituents, we removed irrelevant features prior to the application of machine learning methods. We removed four tissues dominated by immune cells (adrenal gland, salivary gland, spleen, and thyroid), seven gender-biased tissues (breast, cervix uteri, fallopian tube, testis, ovary, vagina, prostate, and uterus), and three hemopoietic related components (blood, bone marrow, and blood vessel). In total, 810 tissue-specific genes representing 16 types of tissue with TSS > 2 were included (Table S1). We used the 101 normal exLR-seq samples to further reduce the number of candidate TSGs: TSGs with a frequency not exceeding 10% in all normal samples were removed. Next, the top n significantly expressed genes for each tissue type were selected and merged into a matrix covering a total of 16 tissue subsets. To determine the optimal n, the system was run iteratively to identify the signature matrix with the minimal conditional number. The signature matrixes with a lower conditional number would be more tolerant to the variation of input expression profile. Finally, 95 genes were included for the 16 solid tissue subsets to constitute the representative tissue signature matrix (Table S4). To establish the signature matrix of hemopoietic cells, a total of 1289 blood-enriched genes (TSS > 0) from the GTEx portal were used to construct the representative blood signature matrix. The hemopoietic specific genes with frequencies > 0.1 among all normal exLR-seq samples were retained. We download the sequencing datasets from seven types of isolated hematopoietic cells and further filtered blood cell specific genes as described in the aforementioned strategy. Finally, 726 genes were selected for seven blood cell components to construct a reliable blood signature matrix (Table S3).

#### 2.2.2 Model selection and construction

The EV-origin is a deconvolution strategy to identify the relative and absolute fractions of blood/tissue sources from exLR-seq profiles based on the hypothetical condition that the exLR-seq admixture is a linear fit of the TSGs that are highly expressed from all the included cell or tissue types. The concept of EV-origin deconvolution is to find the optimal solution of a convoluting equation expressed as AX = B, where A is the transcriptome mixture of the exLR-seq profile, B is the comparable signature matrix for the expression of genes in all types of subset, and X is the vector of relative/absolute proportions of all cell/tissue components.

Additionally, with a final filtered nu-support vector regression (ν-SVR) model, our goal was to investigate a hyperplane that fits as many data points as possible within an optimal distance. Three main steps were included in our EV-origin process. The first step was the zero-mean normalization of the input exLR-seq expression data. The second step was the parameter selection. The ν -SVR model with a linear kernel was tested with different values of ν (ranging from 0 to 1). The parameter with the lowest root mean square error (RMSE) was kept for variable shrinking and model construction. Finally, the relative/absolute proportions of tissue-cellular components in each sample were calculated based on these optimized parameters. All exLR-seq profiles of normal samples were uploaded on the xCell web tool (https://xcell.ucsf.edu/) for enrichment analysis of 64 immune and stromal cell types. The hemopoietic components were collected and compared with the results of the six models in this study.

EV-origin also calculated an empirical P-value for the deconvolution problem using Monte Carlo sampling method [40]. This approach allows EV-origin to test the null hypothesis that the given exLR-seq profile was fully enriched by unconcerned EVs and no identified cell types in the basis matrix (e.g., hemopoietic matrix) are present in a given plasma exLR-seq mixture. For this purpose, we used the Pearson product-moment correlation R as a statistic index calculated between GEP mixture and the estimated tissue-cellular fraction results. This procedure was iteratively tested by gene expression profile in 500 times to generate the empirical P-value of correlation R for each exLR-seq sample. The significant hypothesis-testing result proves that the exLR-seq sample is suitable for EV-origin processing and is not susceptible to the influence by other unrelated components. We have packaged this computational method into EV-origin's core algorithm and uploaded the source code accordingly.

The distribution of estimated liver fractions in different sample groups was tested by Wilcoxon Rank Sum test after using the Shapiro-Wilk test to determine the data normality. Statistical analyses were two-sided and a P-value < 0.05 was considered statistically significant. The core code of our model was written in R script (version 3.6.1) and is available on GitHub (https://github.com/HuangLab-Fudan/EV-origin).

## 3 Results

### 3.1 Overview of the EV-origin approach

**Fig. 1:**
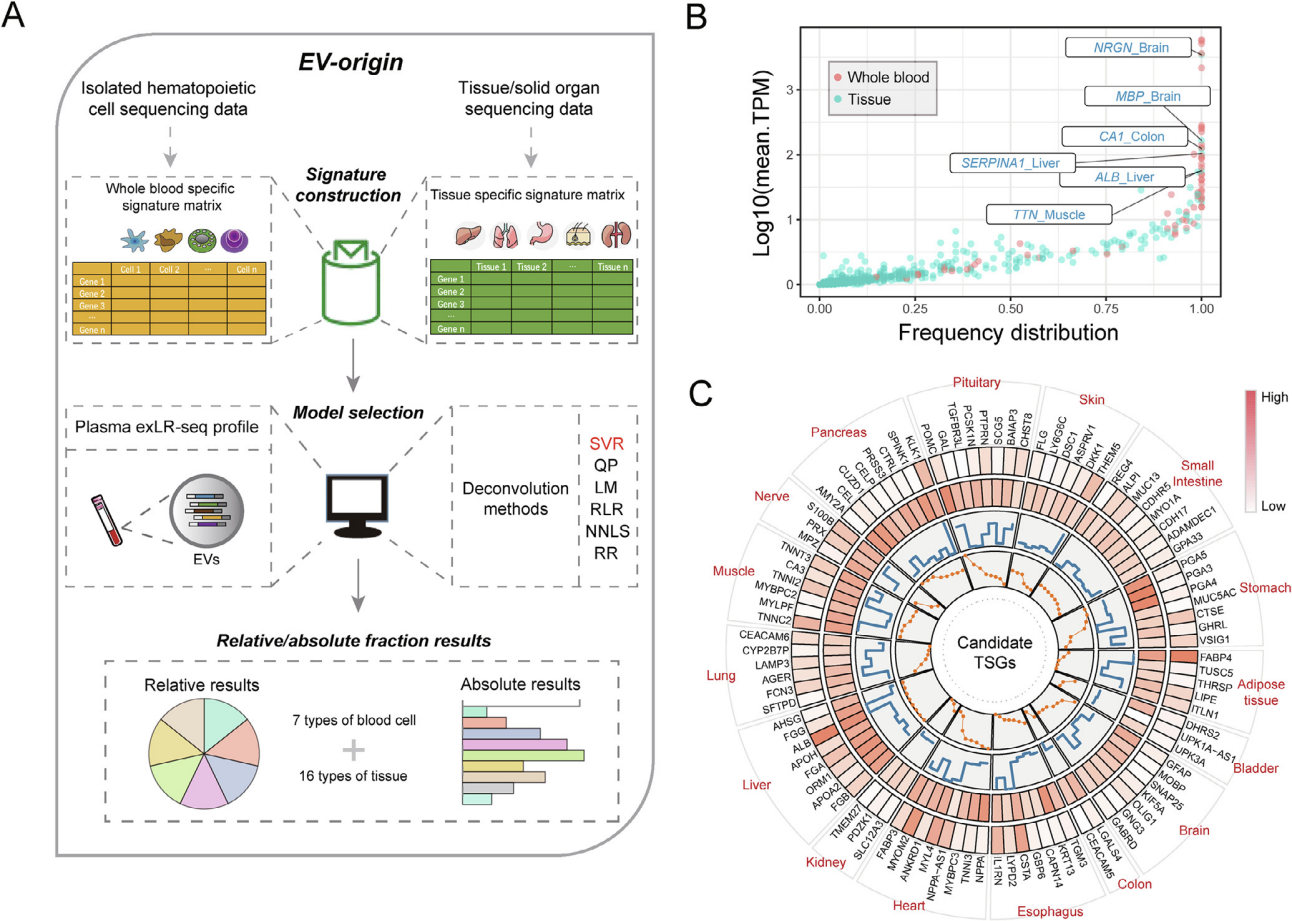
**Schematics of estimating EVs origin from exLR-seq expression profile.** (a) An overview of the computational model of EV-origin. (b) The expression pattern and frequency distribution of 810 TSGs among all healthy exLR-seq expression profiles. (c) The circular figure of 95 candidate TSGs represented 16 types of tissue/visceral organs in EV-origin approach. The circularized diagram was developed by five tracks. The first track integrally identifies the official gene ID of all tissue-specific targets with their represented tissues. The second and third tracks represent the expression level of these TSGs in plasma EVs and tissue respectively. The expression frequency and TSS value of each target are illustrated in the last two panels.

Four main steps were used to construct the EV-origin approach (Fig. 1a, see Methods for more details). The first step was tissue-cellular RNA-seq data processing. We downloaded the raw tissue-cellular RNA-seq data and processed them into a comparable expression profile. The second step was the construction and optimization of the signature matrixes. The TSS strategy was used to filter 726 genes encompassing seven subsets of blood cells (Table S2). We identified 95 genes representing 16 types of tissue (Fig. 1c). These tissue- and blood cell-specific genes were used to build blood and tissue signature matrices. The third step was model selection and evaluation. Six deconvolution algorithms (SVR, NNLS, QP, LM, RLR, and RR) were used to deconvolute the tissue and blood fractions of EVs origin based on two candidate signature matrices. We further assessed the robustness and availability of candidate models using different types of experimental and simulated datasets. The SVR method was finally selected as the core algorithm of EV-origin. The fourth step was to explore the atlas of EV origins from normal or disease samples by an identified algorithm.

### 3.2 The signature matrix construction

To produce the two represented signature matrices, target genes were filtered and selected through four steps (detailed in Methods): (i) raw data collection and pre-processing, (ii) calculation of TSS for each gene, (iii) signature matrix interaction and gene selection, and (iv) validation of the two candidate matrices on validation datasets.

First, we used TSS ranging from 0 to 5 to quantify the tissue-specific property of each gene in each tissue/cell subsets from tissue datasets. The 810 genes with TSS > 2 were regarded as tissue-specific targets (Table S1). We further investigated the expression pattern of this candidate gene set on plasma circulating exLR-seq profiles (Healthy cohort, n = 101, table S5). The blood-specific genes were highly expressed in most of the samples from healthy individuals, whereas the TSGs were expressed in low levels. Notably, we found a much higher fraction of six tissue-specific transcripts from four tissue types were captured in circulation than from other types of tissues (Log10[TPM] > 1.5, frequency = 1; Fig. 1b). Meanwhile, we downloaded the isolated cell sequence data to filter the hemopoietic cell-specific genes and acquire a blood cell signature matrix by the same strategy described above (Table S2). We conducted interacting calculations to make the candidate matrices more tolerant of the variation of expression profile. In total, 726 blood-specific and 95 tissue-specific genes were included to separate seven blood cell subsets and 16 types of tissue components for plasma exLR-seq profiles. These two robust signatures resolved the traceability problem of blood and tissue fraction for circulating EVs (Fig. 1c, Table S3, and 4).

We next examined experimental datasets to further evaluate two signature matrices for the source estimation of the pool of plasma exLRs. Seven isolated blood cells (six platelets and one PBMC sample) and four circulating EVs (plasma with paired serum) samples were used to validate the performance of EV-origin in predicting the platelet fraction. Using the hemopoietic matrix, the EV-origin accurately separated the platelet fraction in isolated cells and EVs profiles (Fig. S1a and b). We also tested the capability of the tissue matrix to predict the brain fraction of EVs in two CSF samples (Fig. 1f). The platelet and brain fraction results from experimental datasets remained accurate in matching the real source of samples.

### 3.3 Model assessment and building

**Fig. 2:**
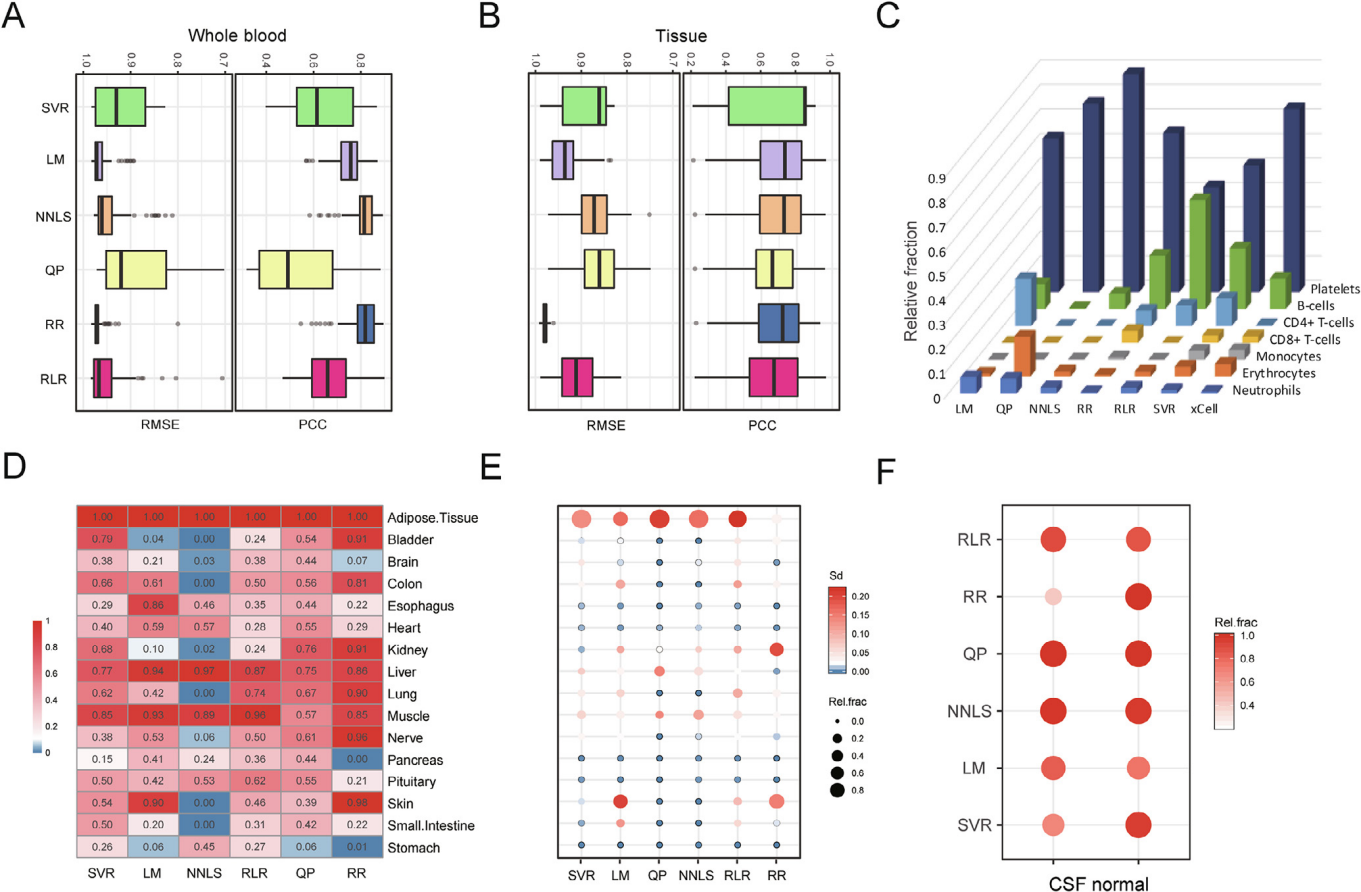
**Comparison of the performance of six deconvolution models using different EV transcriptomic datasets.** (a-b) Root mean square error (RMSE) and Pearson’s correlation coefficient (PCC) between the predicted matrices and input profiles were used to evaluate the performance of the six models on blood and tissue origin of EVs. The higher PCC and lower RMSE of assessed results for each model were respectively indicated to a higher concordance and lower differences between known and estimated tissue/cell-type proportions. (c) The estimated proportion of blood cell components generated from xCell and the six deconvolution models were compared among all normal samples. (d) Evaluation of frequency distribution of relative enrichment results from the six models. (e) Comparison of the stability and relative enrichment distribution predicted by the different models. (f) The comparison of relative prediction results of brain components from each model from normal CSF exLR-seq profiles.

We next benchmarked the six machine learning models on normal exLR-seq mixtures with unknown compositions. The six deconvoluting algorithms were implemented to find the best solution in predicting blood and tissue components with two candidate signature matrices, including SVR, LM, QP, NNLS, RLR, and RR. To compare the performance of linear fitness and estimate the stability of the six models, the concordance between each exLR-seq original expression and inferred mixture was determined by Pearson correlation coefficient (PCC) and root mean squared error (RMSE). The SVR, NNLS, and RR algorithms enabled accurate prediction of the results of hemopoietic components on all normal samples. The SVR method displayed the highest performance in predicting tissue components (Fig. 2a and 2b).

We next explored the frequency distribution of all measured results of the six models among 101 normal samples. We compared the six models with another online cell fraction prediction approach (xCell) in enumerating hemopoietic cell components from normal exLR-seq profiles (see Methods). As illustrated in Fig. 2c, the relative fractions of the six models agreed with those obtained in the xCell approach. Notably, the xCell results accurately matched our results generated by SVR, NNLS, and RR, although there was a slight difference in estimating the B cell and platelet components (Fig. 2c). The fraction results of the SVR and RLR models covered all tissue components with a frequency of occurrence > 0.1 (Fig. 2d). We further evaluated the degree of variance in the component prediction results among normal samples. The SVR algorithm covered all subsets with robust estimation results compared with the other included models, especially for predicting tissue fractions (Fig. 2e). Further, we examined exLR-seq data of two CSF samples to evaluate the power of candidate methods in extracting tissue fractions. Our results showed that NNLS, SVR, and QP were efficient in tracing brain fraction from the CSF EVs samples (Fig. 1f). The predicted results of all subsets from SVR algorithm were hihgly correlated between two replicate exLR-seq samples (Fig. S1c).

We next assessed the detection limitation of the SVR model for rare cell subsets in bulk exLR-seq data. A simulated dataset of one plasma sample with increased cerebrospinal fluid (CSF) content (see Materials and Methods) was used to test the specificity of SVR on complex exLR profiles. The predicted brain fractions were consistent with the actual spiking proportions even when the proportion of the CSF content reached 95% (Fig. S2a). The findings provided solid evidence that the SVR model had high specificity in separating a complex exLR-seq admixture. Based on these comparison results of the included models, the SVR algorithm was finally demonstrated to be a representative computational approach for further exploration of plasma EVs traceability.

### 3.4 The atlas of circulating-EV origins in normal plasma samples

The EV-origin approach was used to resolve the constitution of blood and tissue components of EVs across 101 normal plasma exLR-seq samples. The relative and absolute composition results of seven types of whole blood cells and 16 types of tissues were obtained from each exLR-seq sample. To evaluate the consistency of sample estimation of EV-origin, the result of the absolute fraction of normal samples was analyzed by the t-SNE method [41]. The two-dimensional visualization indicated that the component profile clustered the normal samples into an independent group, with only a few numbers of the sample as outliers (Fig. S2b). We removed eight exceptional samples and the resulting 93 normal samples were selected as normal cohort for subsequent analysis.

**Fig. 3:**
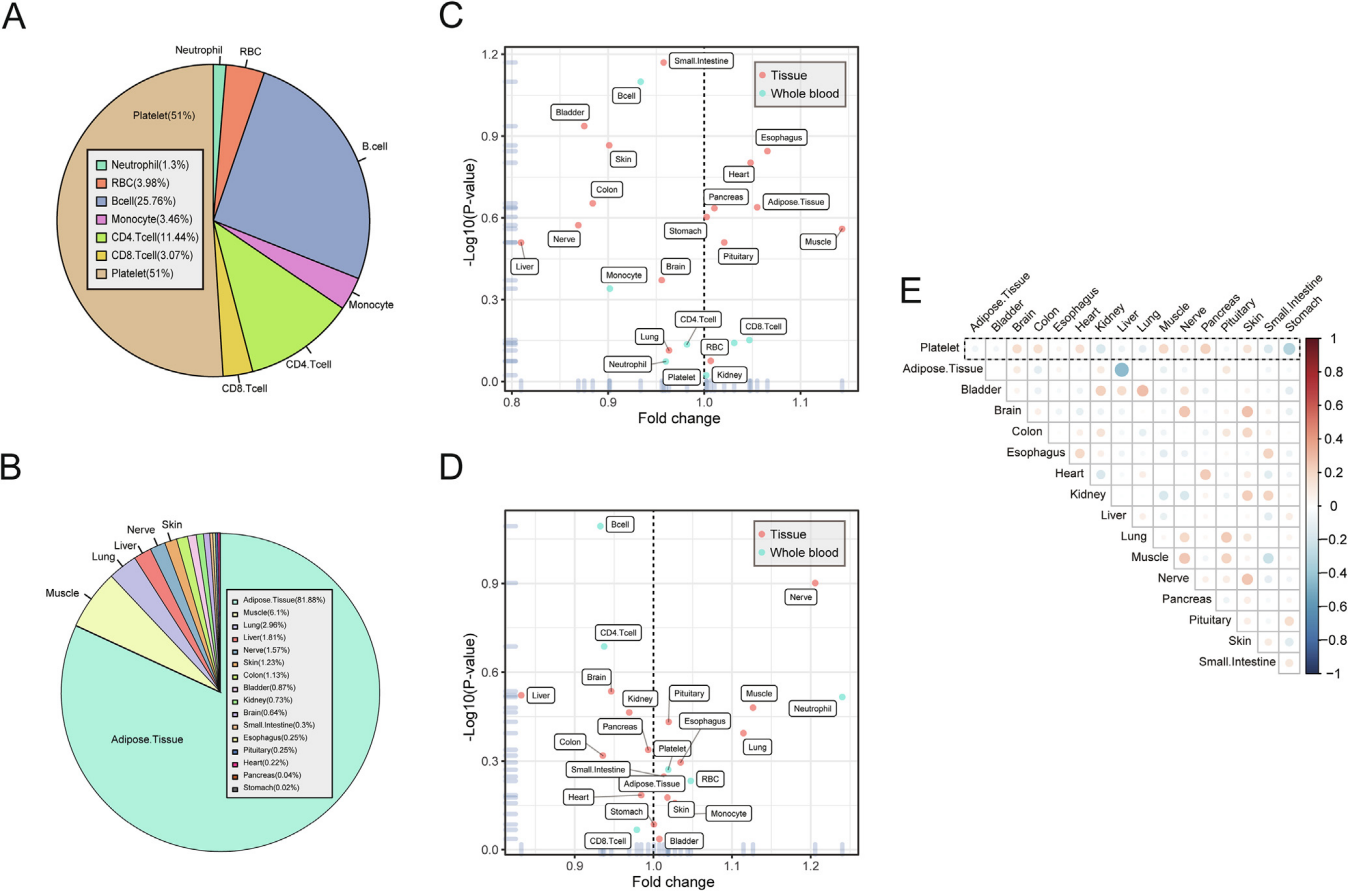
**The landscape of tissue/blood cell origins by the EV-origin approach among normal plasma samples.** (a) The relative distribution of all blood cell components from EV-origin. (b) The pie plot shows the relative comparison of and 16 types of tissue fractions from EV origin. (c and d) The estimation of age-related (c) and gender-related (d) components from EV-origin. (e) Pearson’s correlation between the platelet component with other tissue fractions from the absolute results of EV-origin.

For hemopoietic component estimation, plasma EVs predominantly originated from platelets (51 ± 6%), followed by B cells (26 ± 5%), CD4 + T cells (11 ± 2%), and others cell types (Fig. 3a). Concerning the distribution of tissue constituents, adipose tissue predominated (82% ± 13%), followed by muscle (6% ± 6%), lungs (2% ± 4%), liver (2% ± 8%), and others (Fig. 3b). Adipose tissue was the most prominent tissue contributor to plasma EVs. These exLRs may produce by adipose cells in the hematopoietic system (especially for medulla ossium flava) [42]. We combined the two reference matrices and reconstructed a new signature matrix to estimate the total amount of blood and tissue components. The results showed only 0.2% of plasma EVs were derived from tissues, with 99.8% of them generated from hemopoietic cells.

We then explored whether tissue/cell components correlated with demographic factors of healthy individuals. Differential expression analysis was used to identify the age- and gender-related fractions in normal samples. Notably, none of the blood or tissue fractions of EV-origin were associated with human age and gender (Student’s *t*-test, P < 0.05; Fig. 3c and 3d). These results also indicated that the reliable TSGs were included in EV-origin, which made our prediction results more suitable for exLR-seq samples with different demographic factors. Furthermore, correlation analysis between the platelet and tissue absolute fractions indicated that our approach independently filtered the tissue and hemopoietic components without cross-interaction (Fig. 3e).

### 3.5 Heterogeneity of circulating-EVs origin in diverse disease status

**Fig. 4:**
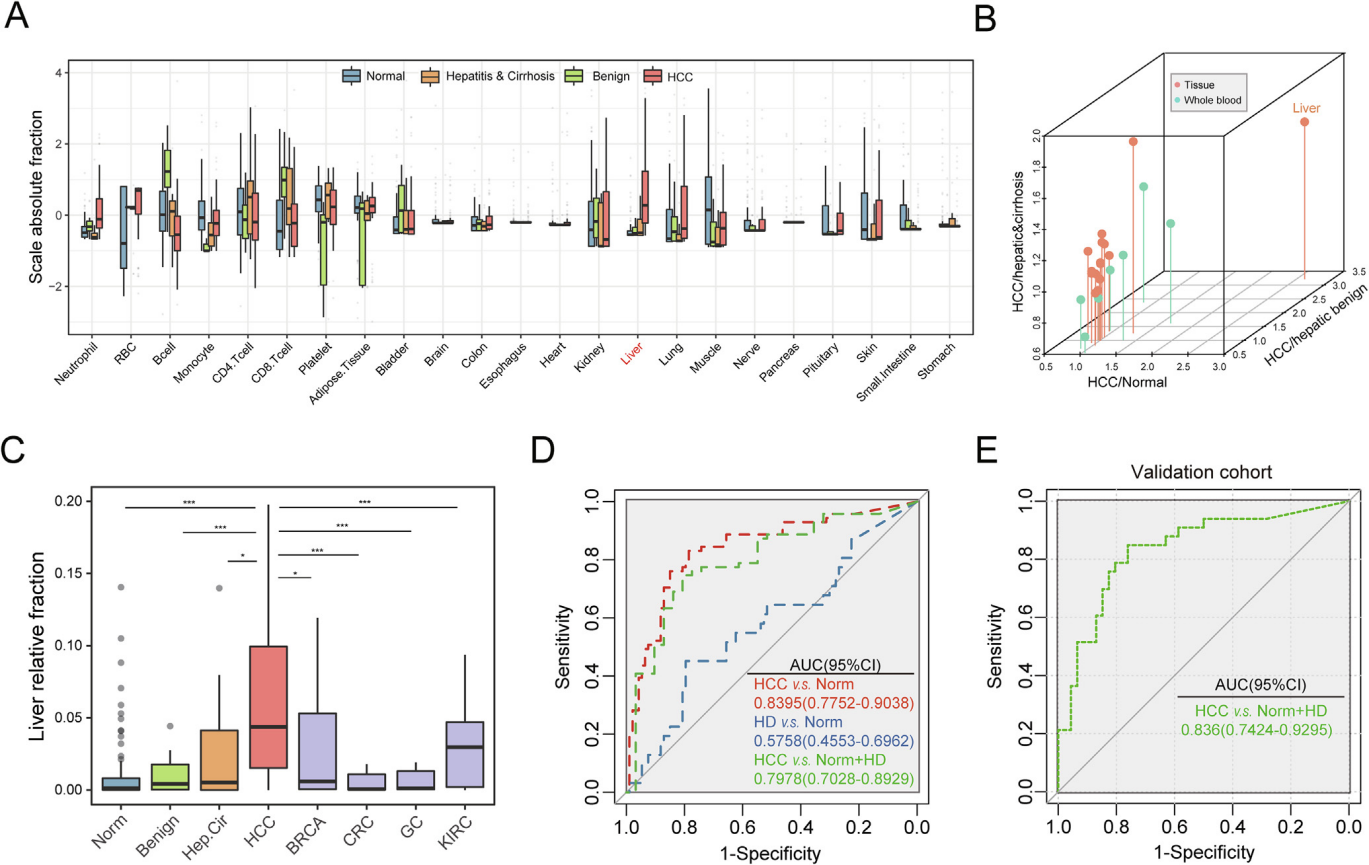
**Heterogeneous origins of plasma EVs from different disease conditions.** (a) Boxplots show the comparison of the absolute liver fraction of EV-origin in diverse hepatic diseases. (b) Three-dimensional scatterplot visualization displaying the fold-change comparisons of EV-origin absolute results for HCC and other types of non-tumor samples. (c) Comparison of relative liver fraction of EV-origin from all types of exLR-seq samples. The results are shown as the mean ± SEM. *P < 0.05; ***P < 0.001. (d) ROC plot unravels the diagnostic performance of EV-origin in distinguishing HCC and hepatic disease samples from healthy individuals. The high diagnostic performance of EV-origin for separating carcinoma from non-carcinoma individuals is also indicated. HD: hepatic disorders. (e) ROC diagram shows the diagnostic efficiency of absolute hepatic fraction derived from EV-origin in validation cohort.

We further explored the heterogeneous pattern of the source contribution of plasma EVs under different disease conditions. The predictive value of absolute fractions was compared among normal and three types of hepatic disease samples (benign hepatic disorder, hepatitis and cirrhosis, and HCC). We have enrolled eight of liver-specific genes (*FGB*, *APOA2*, *ORM1*, *FGA*, *APOH*, *ALB*, *FGG*, and *AHSG*) which were expressed both in liver tissue and normal exLR-seq samples (Fig. 1c). An increasing enrichment of the liver fraction was distributed in samples with liver disease compared with healthy individuals (Fig. 4b). The hepatic constituents were specifically enriched in HCC exLR-seq samples compared to samples from individuals with hepatitis, liver cirrhosis, and other hepatic benign tumors (Fig. 4c, P-value < 0.05). The abnormal enrichment results of the liver fraction of plasma EV-origin may reflect the development of liver damage and the progression of hepatic disease (Fig. S2c). The liver component from EV-origin could also distinguished HCC patients from non-tumor individuals and from individuals with other types of cancer (Fig. 4d, P-value < 0.05). We additionally performed a receiver operating characteristic (ROC) analysis integrated with component results to investigate the potential of EV-origin with respect to disease types. The liver absolute component from EV-origin effectively distinguished HCC patients from non-cancerous individuals with an area under the ROC curve (AUC) of 0.7978 (95% CI: 0.7028–0.8929). In addition, the EV-origin was also accurate in separating HCC from healthy samples, whereas exhibited lower diagnostic accuracy for HD patients (HCC: AUC = 0.8395, 95% CI: 0.4553–0.6962; HD: AUC = 0.5758, 95% CI: 0.4553–0.6962; Fig. 4d).

Consistently, we have tested our EV-origin method in a consecutive validation cohort which includes 40 healthy individuals, 6 benign and 33 HCC patients (Table. S5). Comparing with normal sample, the liver originated fraction was highly enriched in hepatic disease group (mean absolute fraction: control group = 0.007, hepatic disease group = 0.125, P-value = 1.11e-07, Wilcoxon rank sum test, fold-change = 17.49; Fig. S3b). Of note, the predicted absolute liver constituent of EV-origin could effectively separate HCC patients from non-cancerous individuals with an AUC of 0.836 (95% CI: 0.7424–0.9295, Fig. 4e). Moreover, the results showed that the enrichment of hepatogenic fraction of plasma EVs for HCC patients was significantly correlated with clinically accepted biomarker AFP concentration (ng/ml, P-value = 0.038, R = 0.25, Pearson correlation analysis). These results made strong evidence that the EV-origin algorithm has potential application to process hepatic disease identifying and early screening.

## 4 Discussion

Recent studies have revealed the abundance of cell-type-specific RNAs in EVs originating from tissues [19], [43], [44], [45]. A few tissue-specific RNAs were enriched in plasma EVs, reflecting the biological activity and metabolic status of their host cells [19], [20], [39]. Several studies reported the elevated expression level of tumor-specific genes in the circulating EVs and implicated these as potential biomarkers for cancer diagnosis [19], [39]. However, the relative abundance of the tissue cells that are sources of EVs in circulation has remained unclear. In the present study, we optimized an digital approach termed EV-origin to clarify the cell-of-origin landscape of plasma EVs using the plasma exLR-seq profile. The EV-origin could also separate cerebral tissue fractions from CSF exLR-seq data. To the best of our knowledge, our approach firstly enumerates the abundance of hemopoietic and tissue source contributing to EVs from a complex exLR-seq mixture in body fluid.

The differences between EV-origin and those of similar signature gene-based deconvolution programs are as follows: firstly, we used the TSS method to screen all the tissue-specific targets, rather than conservative differential expression analysis. The approach endowed the full transcriptome with tissue/cell specificity, allowing the complete screening of valuable targets according to a given cutoff. Secondly, instead of isolated cells, the tissue signature was constructed by GEPs from the GTEx portal, which reflects the true metabolic condition and physiological status of each tissue. Thirdly, we filtered TSGs with high expression frequency in our internal normal samples. The signature matrices were subsequently constructed using tissue or isolated cell RNA sequencing data, rather than the expression level from microarray profiles. These strategies reduced the computational redundancies, making our approach more reliable for use on exLR-seq data.

Our approach has a number of advantages. Firstly, it provides the expression pattern of TSGs in circulating EVs and allows the development of tissue-cellular traceability system for exLR-seq profile. Secondly, the approach can respectively estimate the total fraction of exLR origin into blood and tissue subgroups for each sample, and can predict the proportions inherent in both subsets. This reduces the interference of certain exLRs co-expressed in both blood and tissue. Thirdly, the candidate model and reference matrices of EV-origin were validated by experimental and simulated datasets that utilized an optimal strategy to robustly trace the tissue cell components from the exLR-seq profile. We used three types of composition-enriched EV samples (CFS, serum, and plasma with different storage times) to compensate for the lack of exact EV-sorting RNA-seq data. Fourthly, the abnormal results of hepatogenic components from EV-origin may indicate varying degrees of liver damage and may correlate with the development and progression of HCC. Finally, platelets release many EVs during the clotting process [46]. These platelet-derived fractions are involved in formation of blood exLRs pool and may reduce the accuracy in estimating tissue components. The EV-origin platelet fraction can be used to evaluate the changes in the collection and storage process of plasma samples and develop reasonable standards for plasma preparation.

However, the most significant current limitation of EV-origin is the insufficiency of EV flow cytometry results in validating the prediction results. We used other EVs sequencing profile from the samples having well-defined cell origin and specific body fluid as an additional experimental dataset to validate the prediction results for each tissue/cellular component. To make up for this limitation, in our subsequent studies, we will sort and collect EVs from different blood cell sources to obtain plasma EV specific transcriptome data, and these data will be used to train the EV-origin model to obtain more accurate prediction results. Meanwhile, we only included seven major categories of hematopoietic cells to evaluate the blood component of EV-origin. This was done because many types of blood cell enriched genes share similar expression patterns in plasma EVs, which could influence the accuracy and specificity of the prediction results. Fewer hematopoietic estimating results, particularly of immune cell components, could diminish the application scope of immunological application of our approach. Moreover, we only filtered tissue-specific targets from the GTEx portal database without reference to other data sets and research conclusions. This may result in incomplete information concerning the tissue specificity of our candidate matrices. In addition, miRNAs are secreted via extracellular vesicles (EVs), which are released from various cell types with tissue-cellular specificity [47], [48], [49]. While the abundance and expression pattern is different between small RNA cargo and exLRs in plasma EVs [19], [50]. To make EV-origin's results stable and comparable, this study did not use miRNA as an applicable resource for estimating EVs origin, which results in the partial deficiency of tissue specificity information of our predicting results. In particular, we integrated the candidate two signature matrixes into the computational model for totally separating blood- and tissue-derived fractions in EVs. Since a large number of blood cell specific genes in hemopoietic matrix, the deconvolution model may overfit during component tracing, resulting in fewer components of tissue-originated EVs in the predicting results. We will add the sorting data of plasma EVs in subsequent studies and refine the preliminary data obtained in this study.

EV-origin can simultaneously measure the component heterogeneity of multiple tissues and organs in biofluid EVs. If we include a large number of blood samples from healthy individuals and those with various diseases, and construct multiple predicting models, our approach can predict the development and progression of diverse diseases, especially the disorders featuring multiple organ lesions, such as malignant tumors and chronic infectious diseases. EV-origin could be a compelling reference for prognosis and efficacy assessments of disease therapy, in the light of heterogeneity of immune cell components in plasma EVs. Notably, exploring the landscape of immune cell components in EVs, particularly T cells fraction, can help us fully understand the interplay between immune system and diseases and indicate helpful information for improving the outcome of immunotherapy in precision treatment. Meanwhile, our findings provide an extensive repertoire of exLRs in normal CSF samples and suggest the potential application of exLRs as EVs source indicator and noninvasive diagnostic biomarkers for neurological diseases. Our approach also enables the large-scale analysis of abundant exLR-seq mixtures to investigate EVs biomarkers and therapeutic targets in biofluids.

## 5 Conclusion

In conclusion, our study depicts landscape of tissue-cellular source contribution of circulating EVs from healthy individuals and patients with cancer. Our results highlight the advantages of EV-origin in the early detection of diseases and other disorders. We believe that EV-origin will become an important approach in disease diagnosis and risk assessment. Our approach could inform the development of exLR-based applications for liquid biopsy.

## Declaration of Competing Interest

The authors declare that they have no known competing financial interests or personal relationships that could have appeared to influence the work reported in this paper.
