# Supplementary material for: EV-origin: Enumerating the tissue-cellular origin of circulating extracellular vesicles using exLR profile
Source: Comput Struct Biotechnol J. 2020 Oct 14;18:2851–9. doi: 10.1016/j.csbj.2020.10.002 (PMC7588739; doi:10.1016/j.csbj.2020.10.002)
Supplement: Supplementary data 1 [file mmc1.docx]

**Supplementary materials**

**EV-origin: enumerating the tissue-cellular origin of circulating extracellular vesicles using exLR profile**

Yuchen Li^a,b^, Xigan He^c^, Qin Li^a,b^, Hongyan Lai^a,b^, Hena Zhang^a,b^, Zhixiang Hu^a,b^,Yan Li^a,b,*^, Shenglin Huang^a,b,*^

^a^Department of Integrative Oncology, Fudan University Shanghai Cancer Center, and the Shanghai Key Laboratory of Medical Epigenetics, the International Co-laboratory of Medical Epigenetics and Metabolism, Ministry of Science and Technology, Institutes of Biomedical Sciences, Fudan University, Shanghai, 200032, China

^b^Department of Oncology, Shanghai Medical College, Fudan University, Shanghai, China

^c^Department of Hepatic Surgery, Fudan University Shanghai Cancer Center, Shanghai Medical College, Fudan University, Shanghai 200032, China

**Keywords:** circulating EVs; extracellular vesicles long RNA sequencing; tissue-cellular origin; tissue-specific genes

***Correspondences:** Shenglin Huang, Ph.D., [slhuang@fudan.edu.cn](mailto:slhuang@fudan.edu.cn) or Yan Li, Ph.D., liyan927818@163.com. Fudan University Shanghai Cancer Center and Institutes of Biomedical Sciences, Fudan University, 270 Dong An Rd., Shanghai 200032, China. Tel.: 86-21-34777580; Fax: 86-21-64172585.

**Abbreviations:**

CSF, cerebrospinal fluid; exLR-seq, extracellular vesicles long RNA sequencing; EVs, extracellular vesicles; exLRs, extracellular vesicles long RNAs; GEP, gene expression profile; TSGs, tissue-specific genes; TSS, tissue-specific score; TPM, transcripts per million.


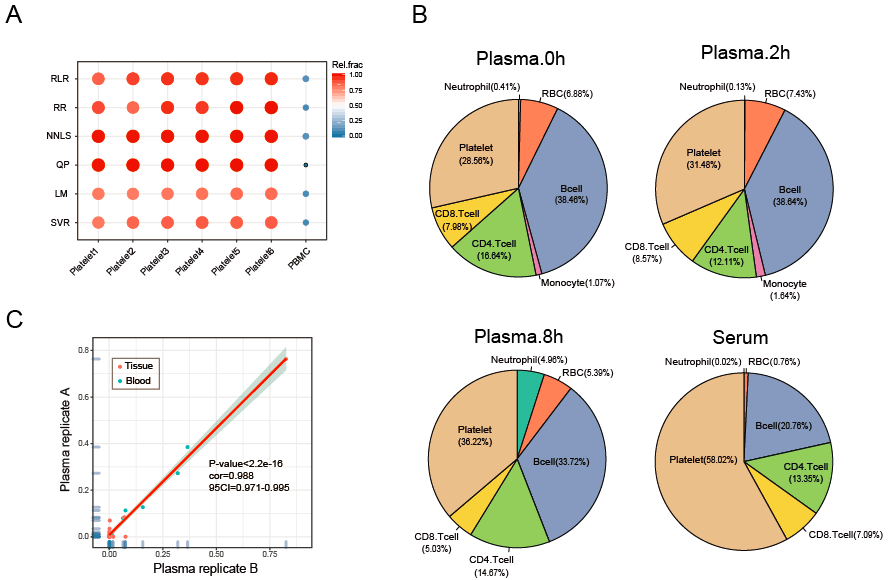


Fig. S1. (a) The predicting results of platelet relative component from all including models in our isolated platelet and PBMC sequencing data. (b) The relative fraction of all estimated hemopoietic components from EV-origin among different types of blood EVs sample (three plasma and one serum sample) from the same individual. (c) The correlation results of all predicting blood and tissue components from two replicate plasma exLR-seq profiles (cor, Spearman correlation coefficients).


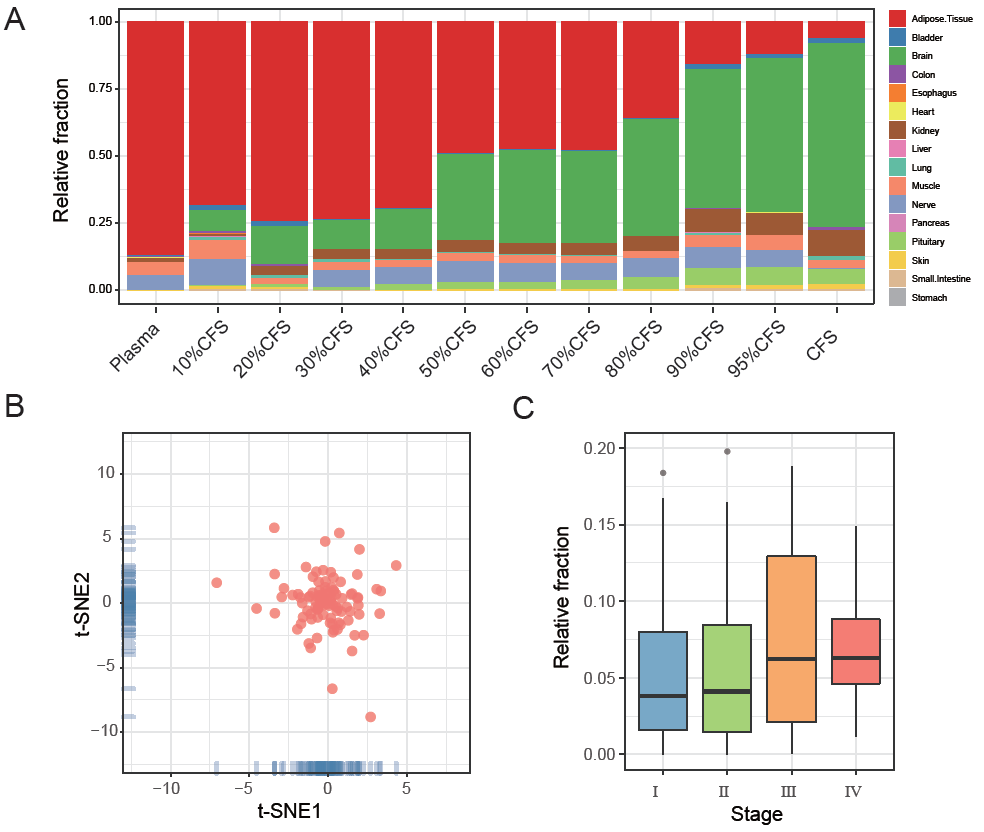


Fig. S2. (a) The model assessment and prediction of EV-origin approach in simulated dataset. The estimated performance was carried out by EV-origin from different synthetical exLR-seq profile comprised increasing level of CSF exLR-seq content. The CSF mixtures were added in ranges from 0 to 95 percent. (b) The two-dimensional scatterplot of t-SNE results for all normal exLR-seq samples. (c) The bar plot indicates the comparison of estimated liver component in different stage of HCC exLR-seq samples. Boxplot lines represent the medians and interquartile ranges of the predicting fractions.


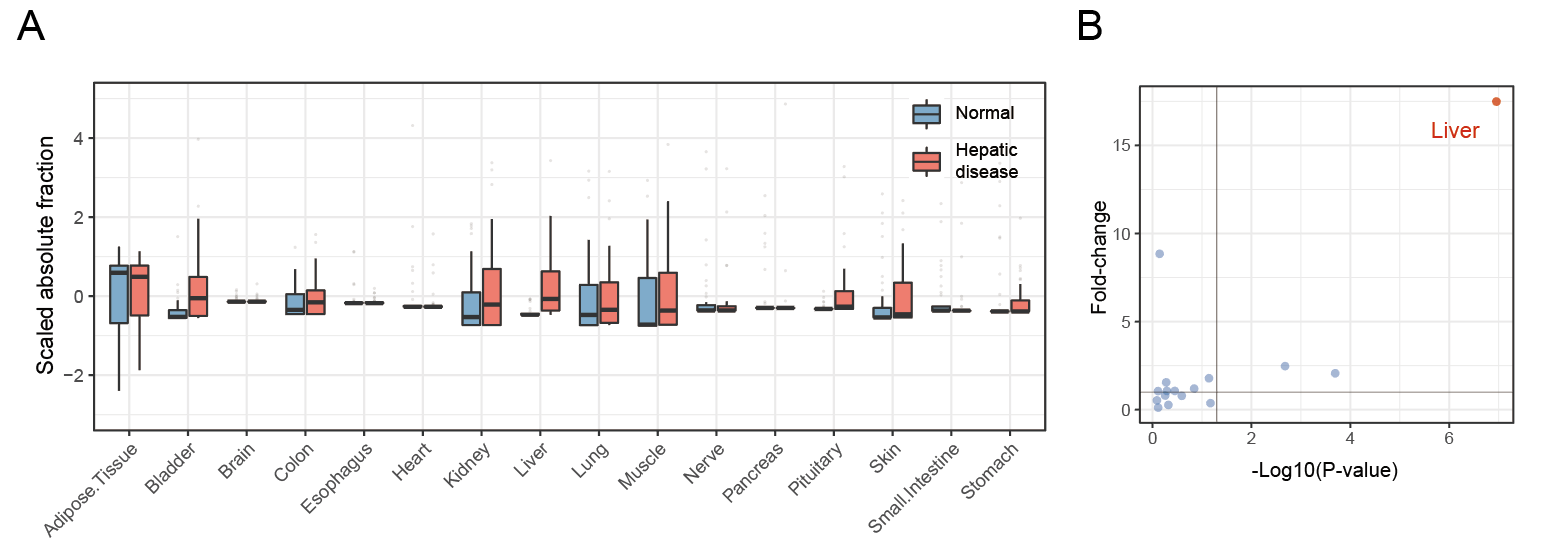


Fig. S3. (a) The absolute predicting results of tissue fractions of EV-origin between normal and hepatic disease samples in validation cohort. (b) The statistical testing results of all absolute tissue fraction between normal and hepatic disease group. The red point refers to the estimated liver fraction (mean absolute fraction: control group= 0.007, hepatic disease group= 0.125, P-value =1.11e-07, Wilcoxon rank sum test, fold-change=17.49).
